# The Influence of Magnetic Field of AMB on Eddy-Current Sensor Operation

**DOI:** 10.3390/s23042332

**Published:** 2023-02-20

**Authors:** Paulina Kurnyta-Mazurek, Artur Kurnyta

**Affiliations:** 1 Faculty of Mechatronics, Armament and Aerospace, Military University of Technology, 00-908 Warsaw, Poland; 2 Airworthiness Division, Air Force Institute of Technology, 01-494 Warsaw, Poland

**Keywords:** eddy-current sensors, active magnetic bearings (AMBs), high-speed machine, control system

## Abstract

This paper presents laboratory results on the influence of the magnetic field of an active magnetic bearing (AMB) on the eddy-current sensor operation. The magnetic suspension technology enables continuous diagnostics and monitoring of a rotary machine and eliminates drawbacks of classical bearing properties. The magnetic bearing system usually consists of two radial and one axial magnetic bearing. It is combined with a control unit, amplifiers and sensors for measuring the instantaneous position of the shaft. For this purpose, eddy-current sensors are frequently used. They operate in close proximity to the electromechanical actuators; therefore, the question arises whether the actuators do not interfere with the correct operation of these sensors. In the paper, the test rig and research plan prepared for that investigation are delivered. Measurement signals were registered from four control channels for different configurations of power supplies for system elements, e.g., with sensors and AMBs turned off, with sensors turn on and at normal work. Recorded time courses are presented and discussed in the paper. For the prepared test rig and AMB/eddy-current sensor configuration, no significant influence of the generated magnetic field from the support is found for the eddy-current sensor output.

## 1. Introduction

In recent years, innovative bearing technologies were developed and implemented in many industrial applications. Both performance and weight of rotating machines, such as compressors, machine tools or generators, are closely connected with the rotational speed of the rotating part of the system [1]. For this reason, choosing the right bearing system has a major impact on the performance of the entire machine. The most common classic rolling bearings are characterized by limitations that reduce the efficiency and a reliability of machines. Namely, these bearings impose permissible values of maximum rotational speeds and limited tribological durability of the rolling elements. This entails the necessity to carry out relatively frequent and operationally troublesome repairs. In addition, rolling element bearings in rotating machines require lubrication systems and additional cooling [2]. A certain improvement on classic rolling bearings is ceramic bearings. They are characterized by lower weight in relation to metallic bearings, which has a beneficial effect on the life of the bearing due to both lower friction and energy consumption by the machine. In addition, the low friction coefficient means that ceramic bearings do not require such frequent lubrication. In addition, they are characterized by a much lower coefficient related to thermal expansion [3]. Another innovative solution in the field of bearing technology is magnetic bearings, which can solve many restrictions and disadvantages of metallic, ceramic and even hybrid rolling rotor–bearing systems during an operational practice [4]. Because of lack of mechanical contact between cooperating elements, they expand the operating range of technical devices and require no lubrication system. Moreover, magnetic suspension has the ability to dampen vibrations and allows the avoidance of friction forces, which cause the heating of elements as well as noise generation. The use of magnetic suspension in its bearing system increases the physical and resting capacity of the motor at high speeds and improves the operating parameters, particularly in periods of sudden, rapidly changing, varying dynamic loads [5,6,7]. To achieve this high performance for devices equipped with active magnetic bearings (AMBs), it is necessary to design an effective controller and high-quality control system.

In the scientific literature, a lot of AMB control methods with dedicated controllers could be found. The most commonly used algorithms are classic proportional-derivative (PD) and proportional-integral-derivative (PID) controllers due to their straightforward implementation [8,9,10,11,12]. Nevertheless, for a high-order nonlinear system and a machine operating in a significantly nonlinear range, the PID control method is ineffective and insufficient. In these cases, the use of an advanced control method such as robust, sliding or even predictive algorithms is preferred [1,13,14]. The mentioned advanced control methods eliminate certain limitations of classic control algorithms and increase the high-speed machine performance. However, enlarging the reliability and precision of the AMB operation is related not only to the control algorithm but also to the quality and compatibility of the entire system.

The magnetic support system of a high-speed machine rotor usually consists of an electromechanical actuator, a control unit, amplifiers and sensors [15,16,17,18,19,20]. The electromechanical actuator is intended to support a high-speed rotor and generate electromagnetic force, providing its stable operation. Further, the control unit should meet the requirements of the real-time system in order to implement a sophisticated control algorithm. Additionally, amplifiers should be characterized by a wide bandwidth. Last but not least, sensors should provide precise information about rotor position.

Non-contact and noise-tolerant sensors are an essential part of control systems in magnetically suspended high-speed machines [17]. Two types of sensors are typically used in the AMB systems, namely induction sensors and displacement sensors. Induction sensors have good potential but are difficult to use because they must be mounted in the bearing air gap [17,18]. On the other hand, displacement sensors are more expensive and introduce some design limitations, but their configuration and assembly are simpler than in the case of induction sensors. For this reason, displacement sensors are typically used in AMB systems [15,16,19,20,21,22]. In typical AMB levitated high-speed machine applications, the displacement of the rotor in the mechanical air gap is measured with commercially available eddy-current-based displacement sensors [16,17,19,20,21,22]. In recent years, these sensors have gained increasing scientific attention [23,24,25,26,27,28] due their wide range of applications in health [29], aerospace [30] and automotive [31] fields. Additionally, improving sensor operational parameters for AMB systems has become an actual challenge for development [32,33,34,35]. In the paper [33], a new kind of differential eddy-current displacement sensor dedicated to AMB systems was shown. In this solution, both the probe and processing circuit are integrated with a magnetic bearing, which results in cost reduction and enables easier application of AMB systems in practice. Moreover, the paper [34] shows a novel circuit design for an eddy-current sensor in an AMB system. It is characterized by compact structure, high sensing quality and low costs. The presented experimental results show that the designed sensor has good linearity and sensitivity, and it can be used in AMB systems. In turn, in [35], a self-differential eddy-current displacement sensor for AMBs used in a molecular pump was presented. In this work, the phase advance network was used to improve dynamic response and resolution of the sensor. The obtained results showed that the designed eddy-current sensor has features of wide bandwidth and high resolution, which improve the AMB system controllability. The mentioned works [32,33,34,35] show that the operation and applicability of the AMB system, apart from a properly selected control algorithm, are also influenced by the measuring sensors used. Their frequency response and accuracy significantly affect the quality of the AMB control system. External disturbances affecting the sensors and their processing systems are also important. Most publications on AMB systems focus on algorithms, but do not consider other aspects of the entire system’s operation, or the interaction of individual system elements with each other and their mutual compatibility. It is obvious that the measuring sensors should work very close to the operation plane of the bearing, especially in the case of sophisticated control systems of flexible rotors. On the other hand, there is doubt as to how close the eddy-current sensor can be mounted so that its operation is not disturbed by the magnetic field of the bearing. For this reason, this article presents the methodology and research results determining the influence of the magnetic field generated by the magnetic bearings on the operation of eddy-current sensors.

The content of this paper is organized as follows: In the first section, eddy-current sensors and the active magnetic support system are introduced and are deeply characterized. Next, the test rig and experimental results are presented. The third section presents a discussion. Finally, concluding remarks based on the achieved results are described.

## 2. Magnetic Support System of High-Speed Rotor with Eddy-Current Sensors

Usually, a magnetic support system consists of electromechanical actuators, the control unit with a controller, amplifiers and contactless sensors. Designing such a system is a complex engineering issue that must reconcile many aspects, including the requirements of safety, precision and speed of operation. Each element of the system has a huge impact on the correctness of its operation.

The basic element of a magnetic support system is the electromechanical actuator. In such systems, four types of actuators could be used, as follows:

- Heteropolar classic electromechanical actuator;
- Homopolar classic electromechanical actuator;
- Homopolar electromechanical actuator with permanent magnets;
- Homopolar electromechanical actuator with electromagnets.

**Figure 1:**
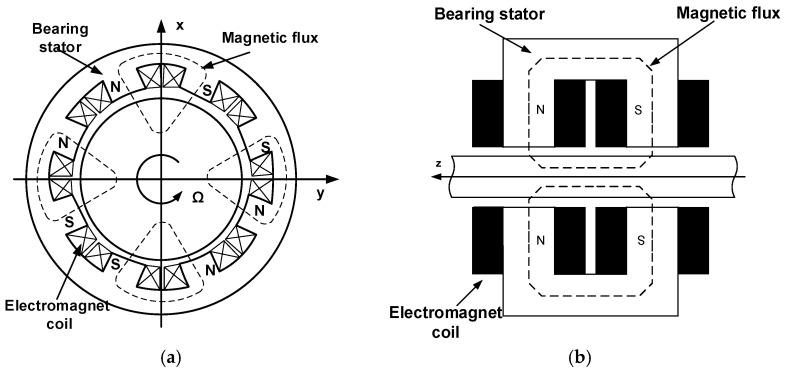
Active magnetic bearing: (**a**) heteropolar magnetic bearing, (**b**) homopolar magnetic bearing.

Classical electromechanical actuators are performed in the heteropolar system and in the homopolar system, which are shown in Figure 1, respectively. In a heteropolar actuator (Figure 1a), any point on the rotor perimeter passes fourfold electromagnet north poles and passes fourfold electromagnet south poles during one complete rotation. Alternating magnetic fields induce eddy currents (a track made of a soft magnetic sheet separated by a varnish layer located on the rotor is required). In a homopolar (unipolar) actuator (Figure 1b), any point on the rotor perimeter passes a pole of the same polarity [36].

Another essential element of the magnetic support system is a control unit, which realizes the control algorithm. In this case, FPGA systems or signal processors are most often used, because they meet the requirements of a real-time system. Control units of the AMBs system usually cooperate with high-resolution analog input and output modules.

Amplifiers are also a part of the magnetic support system. Based on the control signal obtained from the control unit, they ensure proper power supply to the electromagnet coils. Most often they occur as a controlled current or voltage source.

Additionally, a very important element of the AMB system is displacement sensors. Their role is to define the current position of the rotor, which is necessary to determine the correct value of the control signal and appropriate operation of the entire control system. In this case, eddy-current sensors are most often used.

**Figure 2:**
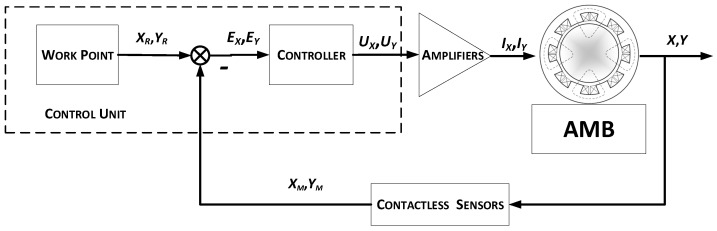
Operation algorithm of single AMB.

The operation algorithm of a single magnetic bearing is shown in Figure 2. The position control process of a suspended rotor follows in two channels, i.e., *X* and *Y*. Contactless sensors generate measurement signals proportional to the current rotor position *X_M_* and *Y_M_*. That signals are passed to the control unit, where based on the control laws and the control error value *E_X_* and *E_Y_*, control signals *U_X_* and *U_Y_* are prepared. In power amplifier control, signals are converted to current control signals *I_X_* and *I_Y_* and supply electromagnet coils [37].

**Figure 3:**
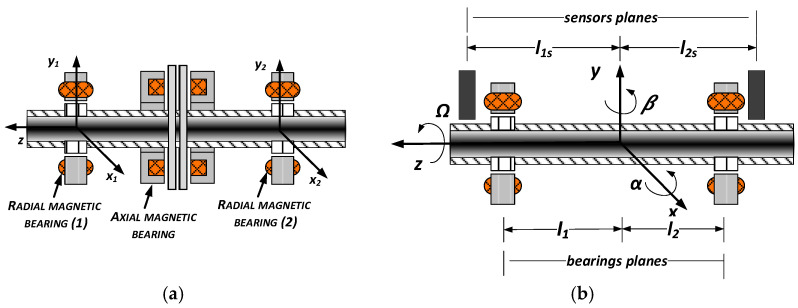
Magnetic support system of high-speed rotor: (**a**) full system scheme, (**b**) operation planes of bearings and sensors.

The magnetic support system of a high-speed rotor should consist of at least two radial magnetic bearings and one axial magnetic bearing, which are shown in Figure 3a. Each radial magnetic bearing generates forces in two perpendicular axes. The first magnetic bearing (1) is controlled in two radial axis coordinates *x_1_* and *y_1_*. The second radial magnetic bearing (2) is controlled in radial axis coordinates *x_2_* and *y_2_*.

Since the rotor position control is performed locally in the planes of the bearings, it is very important that these planes were located as close as possible to the planes of the sensor operation. In this way, the additional calculations involved in determining the position of the rotor in the bearing air gap can be avoided and the control process is more efficient. The operation planes of bearings and sensors are shown in Figure 3b. In this figure, symbols *l*_1_ and *l*_2_ denote the distances from the gravity center O to the left-hand bearing #1 plane and the right-hand bearing #2 plane. In turn, markings with the index “*s*” correspond to the planes of the sensors.

As mentioned earlier, eddy-current sensors are most often used in magnetic support systems. These sensors generate a magnetic field during operation, as well as magnetic bearings. For this reason, it is necessary to verify that these two systems do not interact during operation and do not interfere with each other. The operating principle of eddy-current sensors is presented in the next section.

### 2.1. Eddy-Current Sensor Dedicated to AMB Systems

**Figure 4:**
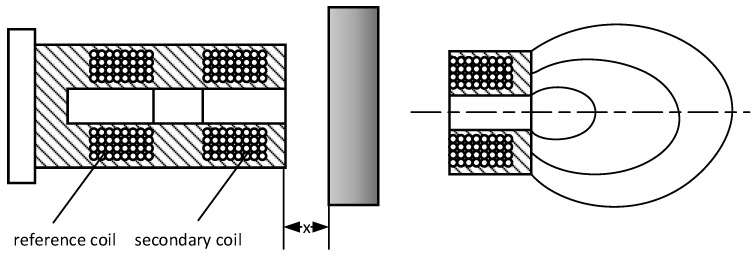
Eddy-current converter.

Typically, in the AMB system application, eddy-current sensors are used to measure the displacement of the rotor supported by means of a magnetic bearing. These sensors can measure the distance of an object made of conductive materials or with a conductive surface, as shown in the Figure 4.

The eddy-current sensor has two coils. The first one is the reference coil, whereas the second one is the secondary coil and is used to detect the magnetic field created by eddy currents in the conductive material. The secondary coil is a solid piece of conductor in the form of a short-circuited turn. The eddy currents generate a magnetic field opposite to the field produced by the secondary coil, which causes changes in the balance between that coil and the reference coil. As a result, the output values of the sensor are the voltage drop on the reference coil with current input or the current drop with voltage input.

### 2.2. Test Rig

**Figure 5:**
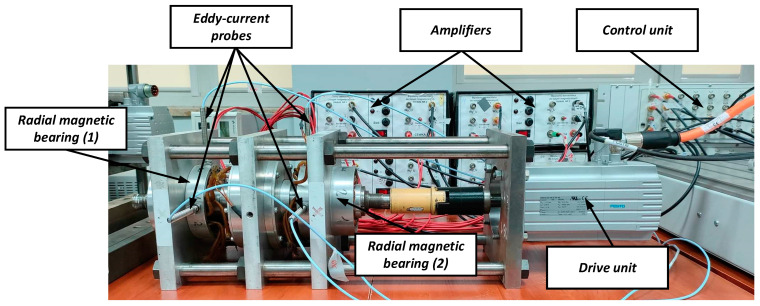
Test rig of magnetic support system.

The magnetic support system of a high-speed rotor, shown in Figure 5, was used for the studies performed in the paper. It consists of two radial and one axial magnetic bearing. The radial magnetic bearings can generate a maximum electromagnetic force of 400 N, and the respective air gap is equal to 0.25 mm. On the other hand, the axial magnetic bearing can generate a maximum electromagnetic force of 600 N, and the respective air gap is equal to 0.3 mm. Additionally, the operating point current for each bearing is equal to 4 A, so the maximum current value available is 8 A [2].

The support system is supplemented by a control system built of the control unit, amplifiers and the eddy-current sensors located in the electromagnet covers. Signals from these sensors provide information about the rotor position for the closed-loop control system and could be useful for a diagnostic system as well. The control unit can be realized on a signal processor or microprocessor. In this paper, the control unit is based on a PC computer and dSpace platform with a signal processor and input/output modules with 16-bits A/D and D/A converters. Control algorithms were designed in MATLAB and Simulink software and then were implemented in the dSpace platform by use of the Matlab Real-Time Workshop (RTW) toolbox.

In the control system of active magnetic bearings, non-contact sensors 3300 XL 8 mm Proximity Transducer System from Bently Nevada were used to measure the instantaneous position of the rotor in the air gap. According to the catalog note provided by the manufacturer, they are intended for static measurements of the shaft position and dynamic measurements during its rotational movement, at which vibrations occur. The measuring system provides an accurate and stable output signal over a wide temperature range. The measuring range of the sensors is 2 mm, with a dead zone of 0.25 mm. For this reason, during the operation of the sensor, the distance between the probe and the measured object should be in the range of 0.25 ÷ 2.3 mm. In turn, the minimum diameter of the shaft whose displacement can be correctly measured is 15.2 mm. The frequency response of the sensors is 0÷10 kHz. Moreover, the maximum values of the linearity deviation and the measurement resolution of the sensor are 1 mils (about 0.025 mm) and 7.87 mV/μm, respectively.

### 2.3. Test Plan

**Figure 6:**
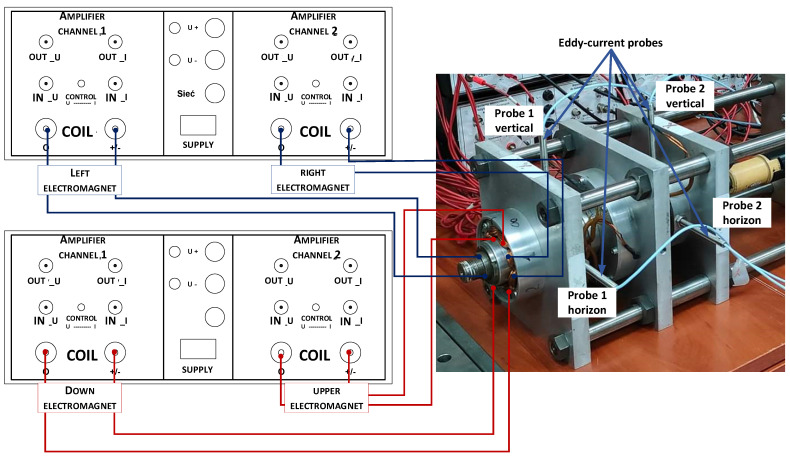
Configuration of test rig for left magnetic bearing.

During the preparation and assembly of the experimental stand for testing, all the manufacturer’s instructions describing the correct configuration of the measurement system were taken into account. Before starting the measurements, the rotor with bearings was placed successively in all extreme positions of the air gap to verify whether it was within the measuring range of the sensor in all cases. This check was made by powering individual electromagnet coils marked in Figure 6 as “Left electromagnet”, “Right electromagnet”, “Down electromagnet” and “Upper electromagnet”.

The labeling of the individual measuring probes and the configuration of the power supply wires of the left magnetic bearing are shown in detail in Figure 6. The right bearing is powered in a similar way (not marked on Figure 6 due to legibility of the drawing).

During the study of the impact of magnetic bearing operation on the indications of eddy-current sensors, the measurement of signals in the time domain from four probes of the eddy-current sensor was recorded in three configurations of the test stand operation:

1. With the power supply turned off for both the magnetic bearings and measuring sensors—measurement noise was recorded;
2. With the power supply turned off for the magnetic bearings and the measuring sensors powered on;
3. Normal operation of the test stand, both magnetic bearings and measuring sensors powered on and working, operating point current equal to 2 A.

The acquired data are presented in the next section.

## 3. Results

**Figure 7:**
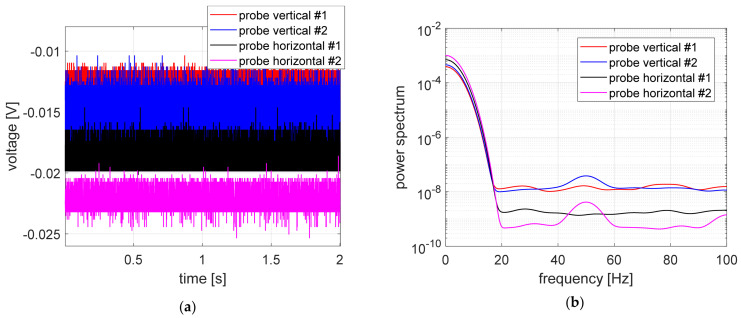
Measurement signals recorded with the power supply turned off for both the magnetic bearings and measuring sensors: (**a**) time courses, (**b**) signal power spectrum.

The measurement results recorded during the first stage of experimental research, i.e., with the power supply to the magnetic bearings and measuring sensors turned off, are shown in Figure 7. The probe labeling corresponds to the markings shown in the legend of Figure 6.

**Figure 8:**
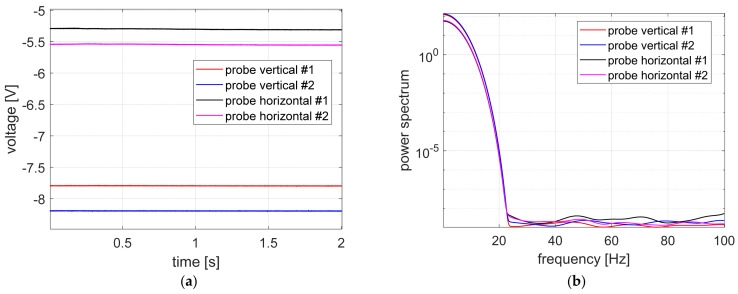
Measurement signals recorded with the power supply turned off for the magnetic bearings and the measuring sensors powered on: (**a**) time courses, (**b**) signal power spectrum.

**Figure 9:**
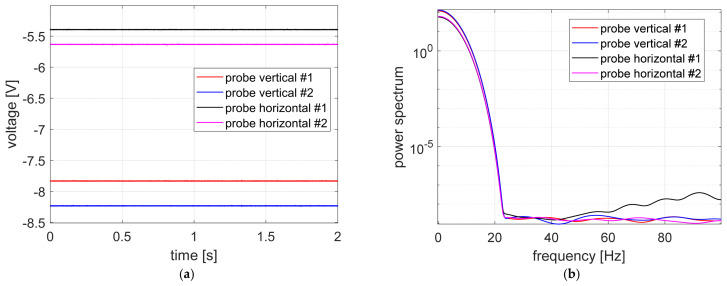
Measurement signals recorded with both magnetic bearings and measuring sensors powered on: (**a**) time courses, (**b**) signal power spectrum.

In turn, Figure 8 and Figure 9 show the time courses and the power spectrum of the signals recorded with the power supply of the measuring sensors switched on and the magnetic bearings switched off, as well as with the power supply of the eddy-current sensors and magnetic bearings switched on. As before, the probe labeling corresponds to the markings shown in the legend of Figure 6.

**Figure 10:**
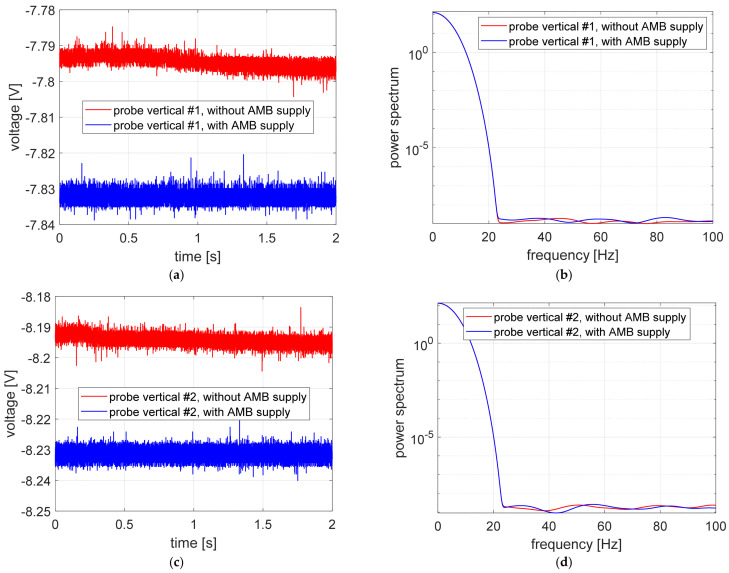

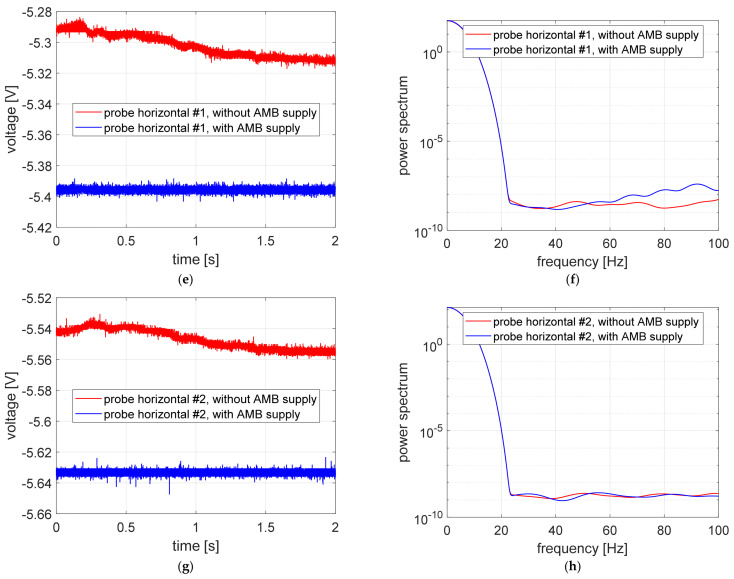
Measuring signals recorded during normal operation of the test stand and with the power supply of the electromagnets turned off: (**a**) time courses recorded by a vertical measuring probe #1; (**b**) power spectrum of time courses presented in (**a**); (**c**) time courses recorded by a vertical measuring probe #2; (**d**) power spectrum of time courses presented in (**c**); (**e**) time courses recorded by a horizontal measuring probe #1; (**f**) power spectrum of time courses presented in (**e**); (**g**) time courses recorded by a horizontal measuring probe #2; (**h**) power spectrum of time courses presented in (**g**).

In order to facilitate the analysis and comparison of the acquired results, the individual waveforms obtained in the same measurement paths were compiled together in one graph, as shown in Figure 10a,c,e,g in the form of time courses and in Figure 10b,d,f,h in the form of power spectrum.

## 4. Discussion

During research, eddy-current sensor indications were recorded under various operating conditions. The measurement was made with the power supply of the sensors and AMBs turned off, with the measuring sensors powered and with the standard work of the system. The signal power spectrum was also determined for each recorded time course.

In Figure 7, the time courses registered during the first stage of studies and power spectrum were presented. All signals had small values ranging from −12 to −24 mV, as expected. Time courses registered in vertical measurement channels had very similar levels.

The time courses obtained by measurement probes for unpowered AMBs are shown in Figure 8. In this case, the position of the rotor located on the lower electromagnet of the bearing was measured by eddy-current sensors. The values recorded in the vertical measurement channels have similar values, equal to ca. −8 V. Furthermore, values registered in horizontal measurement channels also have similar values, equal to ca. −5.5 V. Signal levels in vertical channels are greater than in the horizontal channel because when AMB amplifiers are turned off, the rotor is situated on the down electromagnet, so vertical probes should show the distance of two air gaps. On the other hand, in this case horizontal probes should indicate a value equal to ca. one air gap. A similar relationship between signals registered in vertical channels as well as in horizontal channels is observed in Figure 9, where time courses registered during the third stage of studies are shown.

In order to compare the measurement signals in individual control channels (i.e., vertical #1, vertical #2, horizontal #1 and horizontal #2), the signals registered with the electromechanical actuators switched on and off are presented in Figure 10. For each of the considered control channels, very similar signal values were obtained. Signals registered in the vertical control channels (see Figure 10a,b) are characterized by a more stable indication in the case of the AMB actuator switched off than signals registered in the horizontal control channels (see Figure 10c,d). The discrepancy of the signal values recorded in the vertical control channels is 35 mV, which corresponds to the error equal to 0.4%, while in the case of signals recorded in horizontal channels, these differences are greater and amount to 85 and 95 mV, which correspond to error equal to 1.5 and 1.7%. The maximum error obtained corresponds to a difference of about 12 μm, which is 8% of the air gap value. Values of errors were determined by means of the following relationships:

(1) $$e=\left|\frac{y_{s}-y_{AMB}}{y_{s}}\right|,$$

where *y_s_* denotes the average value of voltage recorded in the case of sensors switched on and AMB switched off and $y_{AMB}$ is the average value of voltage recorded during normal operation.

Comparing the time courses of the measurement signal from the eddy-current sensors recorded with the power supply of the electromagnet coils on and off, it can be concluded that the magnetic field generated by the current flow has no significant effect on the measurement signal obtained, as shown in Figure 10a–d.

## 5. Conclusions

Active magnetic suspensions find more and more applications in various technical devices. They are widely used in rotating machines such as compressors, machine tools or rotary drives. The use of active magnetic bearings allows to monitor the work of the bearing system regularly. Magnetic suspension is also characterized by a greater kinesthetic and resting load capacity, with high rotary speeds and improved operating parameters especially in an emergency, as well as rapidly varying, discrete dynamic loads. To achieve high performance of these system, a well-designed controller as well as high-quality and noise-tolerant sensors are required. Careful selection and installation of sensors in the bearing system is crucial, because it allows to avoid generating incorrect control signals and failure of the entire system.

Due to the possibility of contactless measurement of displacement, eddy-current sensors are widely used in control systems of active magnetic bearing (AMB) applications, especially in the case of the rotor support system of high-speed machine. These types of sensors are characterized by high measuring accuracy of distance changes, simple structure and wide range of bandwidth. Additionally, eddy-current sensors are relatively straightforward in configuration and measuring system commissioning. The main drawbacks are high cost of purchase and the necessity to strictly follow the manufacturer integration recommendations with the system. Research is conducted to miniaturize the sensors as well as achieve embedment of the processing module into its housing. Regarding that, the topic of the influence of the electromechanical actuator operation on the eddy-current sensor output becomes relevant for the whole AMB system functioning. The probe of this sensor generates a magnetic field, just like a magnetic bearing. Hence, the question arises whether these devices do not interfere with their work.

This paper shows the influence of the operation of magnetic bearings on the correct operation of eddy-current sensors. For this purpose, three measurement sessions were carried out, during which measurement signals from four system control channels were recorded. Based on the analysis, it was found that for the presented test rig there are no significant disturbances of sensor indications caused by the operation of magnetic bearings. That confirms a proper design of the system with AMB support and its elements crucial for correct operation. The potential issues, which could occur otherwise, may lead to abnormal system operation, e.g., shift in the working point of the rotor, uneven air gap between the rotor and stator or erroneous control signal generation from the control unit. The dynamic behavior of the AMB support system could be disturbed also, as the control unit will tent to saturate.

The obtained results require confirmation with the use of a higher value of the control current, which will generate a magnetic field with a higher induction value, as well as a position where the distance between the sensor and the electromagnet can be changed. Furthermore, the magnetic field intensity, generated by the AMB, is varying during rotating machine operation, e.g., due to the impact of disturbances and external influences. For that reason, future research activities will be focused on carrying out experiments within various working conditions. In addition, it is worth preparing a model of the bearing and the sensor using, e.g., the Comsol Multiphysics software, for checking whether there is a boundary distance between the sensor and the AMB at a given current, which ensures uninterrupted operation of the sensors. Theoretical calculations can be validated using the laboratory stand presented in the article.

## Data Availability

The data presented in this study are available on request from the corresponding author.
